# Practical Observations on Distortions of the Spine, Chest, and Limbs; Together with Remarks on Paralytic and Other Diseases Connected with Impaired or Defective Motion

**Published:** 1841-07

**Authors:** 


### ART. XIII.

1. *Practical Observations on Distortions of the Spine, Chest, and Limbs; together with remarks on Paralytic and other Diseases connected with Impaired or Defective Motion.* By W. T. WARD, F.L.S. &c.—*London*, 1840. 8vo, pp. 202.

2. *Spinal Curvature, its Consequences and its Cure; illustrated by the History of Thirty-three Cases, successfully treated.* By J. B. SERNY, M.D. &c.—*London*, 1840. 8vo, pp. 90.

3. *Practical Remarks on the Causes, Nature, and Treatment of Deformities of the Spine, Chest, and Limbs, Muscular Weakness, Weak Joints, Muscular Contractions, and Stiff Joints, containing the results of the author's experience, and showing the advantages derived from the modes of treatment which he has recently introduced. With illustrative Plates and Cases.* By J. AMESBURY, Surgeon, &c.—*London*, 1840. 4to, pp. 192.

4. *On a new Operation for the Cure of Lateral Curvature of the Spine, with Remarks on the Causes and Nature of that Disease.* By FREDERIC C. SKEY, F.R.S. &c. &c.—*London*, 1841. 8vo, pp. 50.

5. *Spinal Diseases; with an improved plan of Cure, including what are commonly called Nervous Complaints, and numerous examples from upwards of* 150 *Cases.* By JOHN HENRY ROBERTSON, M.D. &c.—*Glasgow*, 1841. 8vo, pp. 160.

6. *The Cause and Treatment of Curvature of the Spine, and Diseases of the Vertebral column.* By E. W. TUSON, F.R.S. F.L.S. &c.—*London*, 1841. 8vo, pp. 283.

7. *Spinal Affections. A popular Lecture on Disorders and Diseases of the Spine.* By H. C. ROODS, Surgeon.—*London*, 1841. 12mo, pp. 57.

8. *Série de Mémoires sur les Difformités du Système osseux. Avec Planches.* Par le Dr. JULES GUERIN.—*Paris*, 1838-39-40. 8vo, pp. 128, 79, 86, 44, 44, 53.

*Series of Memoirs on Deformities of the Bony System*, &c. By JULES GUERIN, M.D.—*Paris*, 1838-40.

IT was our intention, in collecting the numerous volumes now before us, to give a complete exposition of the very interesting and important subjects to which they refer, especially of the three principal forms of spinal affection: disease of the bones; simple or lateral curvature; and what is commonly termed spinal irritation. For reasons, with which we need not trouble our readers, we must for the present defer this more systematic article, and content ourselves with a short notice of the individual works on our table, gleaning from each, as far as our limits will allow, what strikes us as most useful in reference to the subject to which we shall almost exclusively confine our attention, *Lateral Curvature of the Spine.*

In regard to the treatment of this affection generally, we will only remark that while there are certain general principles which ought always to be borne in mind, there are none which do not often require modification in the attempt to apply them to particular cases. Experience will teach every one as it has taught us this truth. And, accordingly in the treatises before us, some of the authors recommend recumbence as all important, others condemn it, yet instances of cure are related under each mode of management. From facts of this kind it is evident that there are various modes of curing the diseases in question. The more important points to be borne in mind are: the replacement of the vertebræ either by direct permanent or occasional pressure upon them; the removing from them, by recumbency or other means, such force as tends to keep them in an improper relation one to another; the maintenance, if possible, of this due relation by support which, without itself producing ill effects, shall prevent the necessity of confinement to one particular position; and, most of all, the strengthening of the muscles and ligaments which act on the vertebræ by direct means, such as frictions, shampooing, palpation, exercises of various kinds, and by the employment of such general remedies, whether of a medicinal or dietetic kind, as shall best support the health of the system.

I. The volume of Dr. Serny is one of the last published, and probably the most valueless of the lot. It contains cases of various kinds of distorted spines, treated on the plan of the late Dr. Harrison. We do not wish to find fault with this plan, but as Dr. Harrison's book is still in existence we see no necessity for Dr. Serny's production. For those of our readers who may not be acquainted with Dr. Harrison's method, we quote from the volume before us the following:

"The usual mode of cure was commenced by directing the back to be well rubbed daily for half an hour. Compression was then made upon the incurvated spine to encourage its return towards a straight line; a padded shield was then placed upon the right side, extending from the axilla over the brim of the pelvis, and confining it there with stays. A considerable vacancy being in this way established between the person and the shield, the back-bone was observed to move sensibly towards the latter." (p. 33.)

Recumbency constitutes an essential part of Dr. Serny's treatment, while the means to preserve the general health of the system, and to invigorate the muscles in particular, are too much overlooked.

II. The work of Mr. Amesbury is a very voluminous quarto, with an abundance of pictures of crooked backs and limbs, made as straight as they ought to be. The book itself is a sadly meager performance. What is well known about the affections of the spine of which it professes to treat is repeated; but when we seek for information and explanations of the mechanical means which Mr. Amesbury professes to have found of so much advantage, we are referred to this and that patent instrument, the construction of which is not explained, because, forsooth, it may be badly copied. We can give no sanction to this sort of mystification. Genuine professional feelings require a man to tell to his brethren all he knows which can be to their advantage; not to stop short exactly at that point at which his pecuniary profit and their information disagree. It is pleasant, however, to see that Mr. Amesbury has an entire persuasion of the excellence of his own motives, and if this were any measure of truth we should congratulate him thereupon. He very properly insists on the fact, that a treater of spinal deformities should be a surgeon possessed of correct mechanical knowledge; that the mechanist distinct from the surgeon, or the latter without a knowledge of mechanics, is alike unfitted to manage properly cases of this kind. We quote the following remarks as useful hints:

"There are three principles of chief importance to be borne in mind, in the management of all curvatures of the spine, which have not resulted from destruction of part of the spinal bones, or of the substances which lie between them, viz. to bring the bones into their natural position; to sustain them in their natural position by the help of adequate support as long as it may be necessary; and third, to increase the tone and restore and maintain the balance of power in the muscles, especially those connected with the bones of the spine, so that when the natural figure of the bones is restored, the spine may be maintained in its proper direction, by the action of the muscles, without artificial aid." (p. 14.)

It is useless for us to attempt to enter into Mr. Amesbury's plan of treatment. He makes use of certain instruments which, it is asserted, support the spine, whilst at the same time they do not require the observance of a recumbent posture. Whether this be the case or not we cannot determine, as Mr. Amesbury has kept us in complete ignorance of the only means of so doing. We conclude our notice of this book by the following remarks on the mode of *measuring for shoes* and the principles on which this should be done. They appear to us likely to be useful, as, if attended to, they are calculated to prevent the many painful and injurious effects of ill-made shoes:

"Both feet should be measured, partly in the erect and partly in the sitting posture . . . . The first measure should be taken with the person standing upon the measure. The measure round the toes should be taken in the standing posture, with the foot bearing upon the ground. The first gives the extreme length of the foot when the arch has yielded under the influence of the weight; the second gives the spreading of the toes. The third and fourth measures (that is, on a level with the ball of the great toe and about an inch behind this) round the foot, should be taken when the foot is lifted from the ground; all the other measures to be made according to the usual rules which shoemakers are accustomed to follow. The object of this mode of measuring is to cause the shoe to support the metatarsal bones, and to ensure sufficient room for the toes. The support of the shoe should be felt by the wearer, *above the toes and not upon them. The toes should have room to move freely and not be pressed upon by the shoes at any part.* When these rules are duly acted upon by observant persons who have acquired experience in this mode of measuring, the shoes give no more inconvenience to the wearer than a glove, and at the same time gives considerable support to the foot, which is preserved in its natural symmetry and beauty, free from corns and bunions." (p. 191.)

III. We have derived more pleasure from the perusal of Mr. Ward's book than from either of those already referred to; and we will now, with it for our guide, proceed to notice a little more fully than we have hitherto done, several matters of practical importance relating to the general subject of these volumes.

As to the causes of these affections we may enumerate, in the first rank, all those which produce debility, whatever these may be; all causes which tend to encourage a deviation of the spinal column from its normal direction, such as modes of standing or sitting, &c. unequal length of the lower limbs, the use of ill-made stays, pads or dresses which require awkward postures, shortsightedness, inequality of power in the lower limbs, &c. It is needless at the present time to notice the opinion that any curvature of the spine is necessarily associated with disease of its bones. Mr. Ward gives the following table:

"In 282 cases of spinal curvature that have fallen under my observation, and of which I have taken notes, the following has been the proportion in which they have occurred:

| | |
|---|---|
| Of curvature to the right side without disease . . . . . | 230 |
| Of curvature to the left side without disease . . . . . . | 10 |
| Of posterior curvature unaccompanied with disease . . . | 9 |
| Of posterior curvature with disease . . . . . . . | 30 |
| Of that which I denominate incurvation, i.e. projection of the lumbar vertebræ within the pelvis . . . . . . . . . . | 3." |

The same author makes some very rational observations on the power of muscles and the mode of increasing it:

"The comparative power of muscular parts," he says, "depends, 1. On the state of the functions of respiration and circulation, and that increased strength is a consequence of increased vascularity and circulation of blood in a part; and *vice versa*, a want of tone and power of a deficient supply of it. 2. On the degree of exercise or frequency with which they are called into action. 3. On the mental energy or power of solution exerted on them. 4. That the most effectual means of increasing muscular strength is by the frequent exercise of the power itself, and consequently, the preservation of the healthy actions of those functions by which it is influenced. 5. That the muscular parts have a constant tendency to contract, by which they adapt themselves to the state of the limb or parts to which they are attached." (p. 13.)

The bearing of the above principles on the treatment which Mr. Ward recommends in these cases will be sufficiently obvious. And first of all, early in the invasion of the disorder, the diet must be well attended to; the food to be good, plain, simple, nutritious. In young people especially, amusement is recommended to be blended with all the curative means:

"All causes which assist in laying the foundation of the disorder should be avoided; amongst others may be enumerated that of giving strength to one side of the body at the expense of the other, which is done by obliging children to use the right hand exclusively on all occasions, lest they should become left-handed; it is more desirable to increase the power of the arm defective in strength by its more frequent use. . . . . In some slight cases, where I have thought the deviation has been caused by a weakness in one of the ankles, I have recommended the game of hop-scotch, and this has been followed by an evident improvement." (p. 40.)

Mr. Ward points out the necessity of restoring the balance of power between the muscles which have been contracted and those which are in a state of extension. He divides these means into passive and active: the former being all those external means which have the effect of increasing muscular power, such as friction, shampooing, percussion, confinement to a particular position, galvanism, electricity, &c.; under the head of active, the excitement of the muscles by volition or that of muscular exercise. It is unnecessary to enlarge upon the modes of employing and the effect of these various means. Mr. Ward's views respecting recumbency agree much with our own, i.e., that although useful it should not be exclusively relied on, because of the atonic state which the muscles thereby acquire. With recumbency, therefore, must be associated the means alluded to.

"I have witnessed," it is said, "many cases where friction alone has been unsuccessfully employed for a considerable length of time; and others where the inclined plane has been depended on solely, without the other measures being prosecuted at the same time, in which a combined plan of percussion and strong muscular exercise assisted by a recumbent posture has afterwards been attended with complete success. By the union of these means, the cure can be effected in a much shorter time. It would appear that another advantage is gained of great importance with regard to the general health. I have observed that when the recumbent position alone has been relied on, that general ill health and dyspepsia are very often present, sometimes to a distressing degree. This does not occur if recourse be had to occasional action and rest; on the contrary, this regular exercise of the body tends to strengthen the powers of digestion, &c." (p. 47.)

Mr. Ward does not believe that children "grow out of" curvatures, as is sometimes said, unless, indeed, there be an entire change in the habit of life. It should be well remembered that the complaint has a constant tendency to increase unless means are taken to counteract it. The time at which, under the treatment above recommended, some amendment may be looked for depends much on the extent of the deformity.

"The average rate at which improvement takes place, presuming the curvature to be of the extent of an inch and a half or two inches, is half an inch the first month, and one eighth of an inch every succeeding two months." (p. 52.)

There are some very sensible observations in Mr. Ward's book on certain deformities of the chest, and the means to be employed to remedy them. These means are chiefly such as have been already alluded to; such in fact as increase muscular power. The principal deformity here referred to is generally known by the name chicken-breast. Mr. Ward thus describes his own rational treatment of this affection :

"The method which I have employed with regard to the local means in those cases where the spine has been exempt from disease has been that of placing the intercostal muscles and those connected with the anterior part of the chest on the stretch, by placing the patient in a standing position, with the back against a cylindrical piece of wood, and the arms extended backwards. By this means an extension of the pectoral muscles is produced, and they are then brought into full action upon the ribs, as well as the muscles of the abdomen, which are the opponents of them. . . . While in this position the patient is desired to take deep inspirations. I direct manipulation and afterwards percussion to be employed for one or two hours during the day, gradually increasing them in force according to the influence produced on the patient. In addition to these means, I usually desire the patient to suspend the body by the arms, and similar modes of exercise, with a view to promote the full action of the pectorales, serrati magni and postici muscles, &c. on the ribs, to produce the greatest possible extent of elevation of the ribs and sternum, and consequent expansion of the chest. . . . . The good effects of this plan of treatment are not confined to the removal of the local disorder, but it is attended with still more important advantages with regard to the state of the general health. It is uniformly found that in proportion as the parts are restored to their natural form, the pulse is diminished in frequency; the respiration becomes fuller and easier, and the action of the digestive organs as well as of the bowels become more regular and natural." (p. 84.)

In the latter part of the volume there is an application of the same principles of treatment to various other affections, to contractions of the limbs, to paralysis, to St. Vitus's dance. We with pleasure refer to these several subjects, and recommend our readers to do the same, regretting that our limits will not allow us to take a longer notice of them.

IV. Mr. Skey's pamphlet is well written, and contains many sensible and useful observations; but we cannot see that he was called upon to write a book. All the new matter contained in his tractate might have as well occupied a page or two in some weekly journal. He calls attention especially to the fact that lateral curvatures of the spine occur first of all in the lumbar region, and that the dorsal curvature is a mode of compensation for this. We are disposed to concur with him in this view, although unable to determine on the comparative number of cases in which this takes place. He says of this lateral deviation :

" It occupies its general seat in the loins, where it is situated probably in four cases of disease out of five. The lumbar region is by far the most mobile part of the spinal column ; and it forms, as it were, a centre of motion to the whole body. So long as the head is neither inclined to the one side or the other, but retains its natural relation to the trunk, its weight, with that of the trunk, is conveyed in an equal degree by each leg to the ground ; or, in other words, the axis of gravitation occupies the mesial line of the whole body; but no sooner is the position of the head altered, by being carried towards either shoulder, than the lower extremity of the same side receives the additional weight of the head extending over and beyond the axis, in a ratio increasing with the squares of the distances between the projecting head and the axis [?] and if the extension of the head to one side be accompanied by the chest also, then the weight is no longer supported by the two extremities, but by the one belonging to that side towards which the head is inclined, and the other leg is placed on the ground generally in a state of extension, for the purpose of enlarging the base and adding steadiness to the position. In this attitude, an angle is formed at the loins, at which the double lines of obliquity of the body meet; the upper line extending downwards from the head, and terminating in the lumbar vertebræ; the second extending upwards from the foot, which supports the weight of the body, reaching the loins by the same obliquity of direction. This position becoming permanent is all that is necessary to the ultimate completion of true lateral curvature." (p. 45.)

From this view of the subject it is clear that in all cases of lateral curvature, greater attention than is commonly the case should be given to the loins. And it also follows that the proper treatment must be directed to that part, a circumstance not sufficiently attended to in many cases. Having established the great probability of the curvature in a large number of cases having its commencement in the loins, and the consequent necessity of some of the earliest steps of treatment being directed to these parts, Mr. Skey examines the anatomical condition on which depends the permanence of the curve, and suggests a mode of remedying this condition. He states that the longissimus dorsi and sacro-lumbalis are contracted, as must necessarily be the case from the approximation of their points of attachment.

" In order to appreciate the especial efficiency of these two muscles to confirm the injury done, we must examine their structure, and we shall at once comprehend the difficulty of treating lateral curvature, so long as they retain their integrity, even in their reduced condition. . . . . . These muscles are especially tendinous in structure; not indeed like other muscles, in which the muscular fibre terminates abruptly in tendon, which represents the muscle to its insertion, but we have here a layer of tendinous substance of considerable thickness, spread over the surface of the muscle, and into this tendon the muscular fibres are largely inserted. . . . . . . Divide this cord, and the loins become straight in an exceedingly short period of time ; and as the loins present in the large majority of cases of disease the first indication of its presence, and the direct and certain cause of its upper curvature, so it is that the successful treatment of the lumbar occupies the direct path to the cure of the remaining part." (pp. 32-4.)

This division is effected by a "straight bistoury, introduced from the outer side of the muscular pillar, across its extent or cutaneous surface, and with which the surface only of the muscle need be divided." After this the other steps of treatment may be pursued. The main indication for employing this mode of treatment is (when it is rendered unobjectionable by the absence of many well-known causes and conditions of curvature) the tension of the muscles on the concave side of the curve, when an attempt is made to raise the spine. The treatment here recommended by Mr. Skey is also said in his hands to have proved, and still to be proving, very beneficial. Sufficient time has not yet elapsed for any judgment to be safely arrived at respecting the expediency of the operations, of which the above, described by Mr. Skey, is an example. Various muscles have been divided by different surgeons to remedy curvature of the spine. Some have divided muscles high up in the back, which, if Mr. Skey's opinion be correct, must end in failure. But we are not yet in possession of sufficient data to be able to express any safe opinion on the probable result of such operations. But Mr. Skey's proposition, and the reasons in favour of it, well merit the attention of the practical surgeon.

V. We may say of Dr. Robertson's book the same as of Mr. Skey's; it is not a bad book, but it is not needed. It will, however, be useful to those who have read none other on the subject. We are disposed to think that the most useful part of the volume is its author's description of his mode of employing dry cupping (in such cases as he thinks this applicable to a tender spine), and a description which he gives of a substitute for stays. This dry cupping is very much relied on by Dr. Robertson in such cases, whether the spine be curved or not, as there is a cause of irritation at the roots of spinal nerves making itself known by distant symptoms; tenderness on pressure of the spine, or over its transverse processes, being the indication for the employment of the cupping. Having ascertained personally since reading Dr. Robertson's book the powerful local effect which may be produced by cupping, according to the mode described, we think that we shall be doing service by making it more generally known:

"In treating," says Dr. Robertson, "both the serious affections of the back, and those less severe, and depending on irregular muscular action, disturbed nervous action, or pressure upon a nervous twig, the cupping-glasses are the most powerful remedy I have ever employed or seen employed . . . . . I have been in the habit of practising it for upwards of twelve years, in an immense variety of cases; and in the hope that the attention of the profession and of the public may be more directed to it, have given cases illustrative of its efficacy . . . . . . . With reference to the cases where it is likely to be useful, I should say, that wherever the pain is dull though severe, deep-seated, chronic, not much increased by pressure, or has *refused to yield to ordinary means*, there would I put it in practice; and, if required, on a part of the body where a cup would not sit, or the nearest convenient spot." (pp. 70-1).

The following is Dr. Robertson's description of the most efficacious mode of employing his cupping-glasses. Having dipped the glass in warm water,

"A little piece of folded absorbent paper is dipped into alcohol, brandy, or pyroxilic spirit, keeping the end by which you hold the paper dry. Apply this to the flame of a candle, and let it drop to the bottom of the glass to be used. . . . . . . The dry portion of the paper adheres to the wetted or damp portion of the glass; and when the latter is inverted and applied to the skin, the flame remains at the point furthest from the skin, and causes no irritation from its presence. . . . . . . The method described, though not perhaps the most elegant, is by far the most powerful way of producing sudden determination towards the surface, and alteration of the internal action of a part. I can in almost any instance in a favorable part of the body fill the glass nigh half full of integument and muscle, and in a *few* instances have seen the blood sweat through the pores of a healthy but fine skin." (pp. 72-3.)

The cups employed by Dr. Robertson are peculiar, and well adapted to fit themselves to surfaces, to which glasses of the common form cannot be applied.

"Even the largest of the ordinary cups is in most cases too small. . . . The shape of the mouth too of my glass is quite different. Instead of the usual plain round mouth, similar to that of a tumbler or wine glass, I have it cut out at the sides, so as to make the *mouth* of the glass a segment, more or less small of a large circle, both sides being alike, thus, ‿. I consider this a great improvement." (p. 76.)

And we think so too. The glasses employed by the author vary in size from six to twenty ounces. They are capable of producing a very powerful effect, and are well worthy of employment in a variety of cases of local pains. The cases related by Dr. Robertson illustrate their efficacy in relieving pains and spasmodic affections, probably having their source at the origin of nerves in the spinal marrow.

We must also notice Dr. Robertson's description of a "simple and efficient back stay," which he has employed in cases requiring support, but which are injured by ordinary stays; this, he says, he has found to answer admirably.

"A piece of well-seasoned ash, one eighth of an inch thick, two and a half inches broad, and fifteen inches long, was steeped in boiling water, actually boiled or submitted to the action of steam for twelve hours. By this means it became perfectly manageable, and would take easily any shape given to it. It was then covered with chamois, and padded one third of an inch thick in front. Three straps of powerful linen were put across it at certain distances, with buckles, and shoulder-straps also with buckles, the highest strap buckled quite loose above the breasts in the female, the other two at distances lower down. The shoulder-straps came over the shoulders, and were buckled to the first strap already fixed, thus keeping the whole support sufficiently high up, and the upper belt out of the way of the mammæ. . . . . Some have laid aside their expensive steel corsets, and taken to this simple affair; and in no single instance, when made to fit, have I had any complaint, either of its inefficacy, of its producing irritation, or even of its inconvenience." (pp.98-9.)

VI. We wish that we could give praise in proportion to its size and cost, to Mr. Tuson's book. But as may be said of most of the others, two or three pages of a journal would have been the most appropriate vehicle for conveying to the public all the useful matter which it contains. It is true, that we must then have sacrificed numerous pictures of crooked spines and deformed chests, and long histories of cases little worth the reading; that the draughtsman would have been without an opportunity for displaying a very moderate talent for zinc engraving; and, more important than all, that the printer would have had to seek elsewhere the means of exhibiting a very excellent type: but Mr. Tuson would have avoided the publication of a book which scarcely communicates any novel matter which might not have been well compressed into the space occupied by the preface. There is much common-place about the necessity of ascertaining the causes of curvature of the spine before undertaking their treatment; and one is almost led to believe from such a sentence as the following, that the author imagines this to be a discovery:

"I presume that sufficient reasons have been advanced in support of this doctrine, to induce the reader to agree with me on this point; namely, first to ascertain the seat and cause of disease. He will assuredly see further ground for concurrence in this opinion, as I proceed to the consideration of the separate affections to which the spine in so many cases is subjected," &c. (p. 22.)

After this specimen of presumed ignorance on the part of the reader, Mr. Tuson takes great pains to show, what everybody else knows, that a variety of causes may bring about curvature of the spine, and that the treatment must vary in accordance with such causes. It is surely needless to comment on such a matter as this. Mr. Tuson thinks that there is often a softened state of the vertebræ themselves in cases of lateral curvature. He speaks of the benefits derived from proper exercise, food, &c. in the development of bone and other parts; and he tells us that "those of his readers who have observed the growth of youth, must have seen the frame gradually expand when the child has been allowed a proper degree of exercise, with suitable food and clothing, except in the case of some predisposing cause for disease." This is the important mode in which the fact of growth is communicated to Mr. Tuson's "readers." What will happen to the pitiable many who cannot buy this book? But we will, for the information of those who may not be Mr. Tuson's readers, refer to the apparatus employed by him in some cases of curved spine; and which apparatus produces extension of the vertebral column, and affords convenient means for employing certain exercises whilst the recumbent position is observed. Those who wish to employ the instrument recommended, will do well to procure a sight of it, as without diagrams it is difficult to convey a sufficient description of it. The instrument is a couch, on the horizontal framework of which are laid two stuffed cushions, which are acted upon by two springs. The effect of these two springs is to separate the cushions from one another, so that when the patient is lying on the couch, extension may be made both to the upper and lower part of the spine. In order to increase the power of these springs, weights are applied, the action of which may be varied according to the necessity of the case. At the head of the horizontal part of the couch is an exercising framework, with rollers and ropes adapted to various exercises for the patient's arms, and the position of this framework may be altered as necessary. So that "independently of extension, one of the purposes of the couch is to enable the patient to use a variety of exercises whilst in the recumbent position, which will tend to dilate the chest and increase the function of respiration and circulation." There is an abundance of drawings to illustrate the use of this instrument; and there are many other drawings, some well done, some very ill done, showing the healthy state of the spine and its morbid states; but after having reconsidered all the attractions of the volume before us, we are compelled to adhere to the opinion of it above offered.

Some of our readers may think it out of place to criticise the style in which a practical treatise on affections of the spine is written; but we are of a different opinion: the honour and dignity of our profession are concerned in the literary as well as the scientific and practical knowledge of its members. We should like to know what reception a work written in a style of which the following is a specimen, would receive at the hands of a literary reviewer? or what estimate he would form of the education and taste of a profession, one of whose distinguished members could thus express himself?

"With him [the anatomist] as with the astronomer, the laws of nature are fixed and immutable; and he raises his voice against whatever may tend to derange and pervert their natural order or fundamental principle. Before him nature stands unveiled; he investigates with a peering eye that incomprehensible structure, the human frame; he explores the deep and secret recesses of its hidden mechanism; he knows that in the organization of animal life, there are certain fixed laws, which, in whatever species they may be found, are invariably the same, having the same nature and sympathies, the same vital and physical principles, emanating from the same cause, and destined for the same functions." (Preface, p. xi.)

VII. Mr. Roods' little work scarcely demands notice here. It contains nothing new, and professes not to contain anything. It probably was written for the instruction of the members of some Institution, and it answered the purpose very well.

VIII. M. Guerin has been long known as a writer on orthopædic subjects, and as a clever and energetic practitioner; but there has occasionally been too much of display in his proceedings. He is, however, a man of much scientific knowledge of his subject, and of great experience. His first memoir is devoted to the explanation of a new method of curing lateral deviation of the spine, by what he terms sigmoid extension and flexion. Of this he observes that "it rests on two new principles substituted for two old ones, oblique or perpendicular extension employed instead of parallel extension, and flexion instead of compression." The mode of treatment spoken of is applied to all kinds of articular deformities. We may premise that M. Guerin is a most wordy author, and that the kernel of his compositions bears a very small proportion to its coverings, and is at the same time somewhat difficult to extract. In the present instance, it consists of a drawing with a description of a most elaborately constructed bed for producing the desired effects on the spinal column. Any attempt at a description of the mode of construction of this apparatus would be useless without a drawing of the apparatus itself, for which we must refer our readers to M. Guerin's work. The following quotation from the book itself will explain the principle of M. Guerin's method:

"In a mechanical point of view, what is required to be done in the treatment of lateral deviations of the spine? To restore to its proper form a column which is curved in one or two points of its length. . . . . Instead of the spine, take a curved but flexible column. The proper mode of straightening this is not to pull at its two extremities and in the direction of its length, but to fix the two extremities of the column, applying at the same time the knee to the convex side of it, and at the same time pulling perpendicularly at its extremities, thus producing a curve in an opposite direction, and the individual doing this will not extend merely until the column is straight; experience teaches that, in order to effect a complete and permanent restoration, it is essential to produce an opposite curve to that which existed before, in order to destroy the force which tends to reproduce it when the force has only been so far employed as to produce a straight line. This, which is commonly done to get rid of any kind of flexible curvature, is what I have endeavoured to render practicable for curvatures of the spine. The method which I propose consists in substituting artificial for pathological curvatures, directly opposed to the last, so as to give to the column the form of an S in a direction exactly opposite to the S which represents the pathological change; in other words, it consists in substituting oblique and perpendicular extension for parallel extension of the spine. Hence the term *sigmoid*."

If we may judge of the efficacy of M. Guerin's method from his commendation of its effects, it is very useful indeed: and a report made upon certain cases of lateral curvature, which were subjected to treatment with the apparatus, appears to justify the inference that there is considerable advantage to be gained from its employment.

From the second memoir of M. Guerin we learn that simulations of the curved spine are means employed to deceive their fellow-creatures, both by medical men and laymen. It seems that this subject has excited a good deal of strong feeling among some orthopædists in France, but it does not appear to us to be of sufficient importance to employ any space upon it.

The contents of the third memoir does not refer to spinal diseases; but we shall nevertheless briefly notice it. It is on wry-neck produced by muscular contraction, and will be new to some of our readers. It is maintained by M. Guerin that the sterno-cleido-mastoid muscle consists of two distinct muscles, the sterno-mastoid and cleído-mastoid; that the functions of these differ, the cleido-mastoid being a motor of the head, the other a muscle of inspiration; that in wry-neck, the sternal portion of the muscle may be the only part which is contracted, and that the section of this portion alone suffices to remove the essential cause of the deformity. It is not maintained that both parts of the muscle may not simultaneously contribute in some cases to the deformity, but that the cause is most frequently such as, M. Guerin mentions, results from his own observations. There appears to be nothing in the mode of operating which requires description; the object being to render the tendon as tense as possible, and then to divide it beneath the skin. In the other treatment of these cases, the passive retraction of other muscles is to be overcome by proper mechanical contrivances.

The fourth and fifth memoirs are on clubfoot, and are among the most valuable of the series. We have, however, so recently devoted an article to the subject of clubfoot that we shall here omit it; remarking only, that in the fifth memoir will be found some very careful descriptions of the anatomical peculiarities of the tarsal bones in various forms of clubfoot, and with which it is very desirable that operators for this deformity should be acquainted. At the same time we cannot omit to remark on the very tedious prolixity of the composition.

The last memoir in the series is on rickets; and although foreign to the main object of this article we shall here briefly notice it, as we may not have another opportunity of referring to M. Guerin's works. This memoir contains an excellent description of the morbid anatomy of the bones in that disease, which we will abridge. In the early stage (*incubation*) the bone is filled with an effusion of sanguinolent matter, which occupies its spongy tissue, its medullary canal, the space between the periosteum and the bone, the concentric lamellæ of the diaphysis, and all parts, whether of the long or of the flat bones, in which the nutritive arteries are distributed. The swelling of the different parts is the result of this effusion. In the second period (*déformation*), at the same time that the texture of the bone softens, the matter which is deposited in its interstices tends to become organized. This matter is most abundant between the periosteum and the bone, between the medullary membrane and the canal. In the third period (*résolution*) the newly-formed tissue becomes harder and more compact, and is compounded with the old bony structure, which regains its former hardness. This addition of new matter causes the increase in size, which is witnessed in this disease. In an exaggerated degree of the disease (*consomption rachitique*), the interstices of the bones and lamellæ are far separated, and the consistence of the original bone is so far lost that their external layer is often reduced to a thin pellicle. The texture of rachitic bones in the adult, when the disease is completely resolved, is much harder and more compact than in the normal state. In this condition (*eburnation rachitique*) it is no longer possible to distinguish between the original bone and the parts of recent formation.

We have thus shortly noticed the various books before us. From those which treat of deformed spine, a tolerably rational system of treatment may be adduced; and we should rather make a selection from all than attach ourselves exclusively to one. Thus, for example, the advocates of a system which excludes recumbency must, in certain cases where great pain or fatigue attends on any exertion, make recumbency a part at least of their early treatment, until that degree of amendment has been effected which will allow of the adaptation of such well-applied mechanical support as will permit the body to be exercised without the whole weight of its upper part resting on the spine. We can only regret that Mr. Amesbury has not, by a description of his apparatus, put it in our power to judge of its fitness for the professed object. The means referred to and explained by Mr. Ward must be in various degrees applicable to all cases, and with a certainty in many of a favorable issue. We are indebted to Mr. Tuson for the description of his apparatus, which appears to be well adapted to effect extension in the cases in which this is desirable, and which also affords facilities for certain useful exercises. The operation proposed by Mr. Skey may be auxiliary in those cases where the contracted tendon is evidently the cause of deformity, though we do not think that it is quite clear that the muscles referred to by him are the main instruments in keeping up displacement.

The most important practical lesson to be learned from a consideration of the whole subject before us, is that which is derived from a knowledge of the causes of curvatures of the spine. The majority of the more immediate causes would not suffice to produce the disease if their operation was not favoured by a constitutional debility; and this debility is induced in various ways. It is therefore to the prevention of the causes of the deformity that the attention of the medical man should be especially given. The probability of its occurrence in all families, and especially among the female members of them, should ever be borne in mind; and changes, which if allowed to gain ground may become very difficult of cure, may then be anticipated and prevented. More attention than was formerly the case is now given to early physical education; and if this be continued on a wholesome plan, the occurrence of distorted spine must be proportionally rare in future.

